# Polyphenolic Flavonoid Compound Quercetin Effects in the Treatment of Acute Myeloid Leukemia and Myelodysplastic Syndromes

**DOI:** 10.3390/molecules26195781

**Published:** 2021-09-24

**Authors:** Cristiane Okuda Torello, Marisa Claudia Alvarez, Sara T. Olalla Saad

**Affiliations:** Hematology and Transfusion Medicine Center—Hemocentro, University of Campinas, Campinas 13083-878, Brazil; marisacalvarez@yahoo.com

**Keywords:** polyphenolic compounds, quercetin, hematological disorders, anti-proliferative effect, apoptosis, autophagy, epigenetic modifications

## Abstract

Flavonoids are ubiquitous groups of polyphenolic compounds present in most natural products and plants. These substances have been shown to have promising chemopreventive and chemotherapeutic properties with multiple target interactions and multiple pathway regulations against various human cancers. Polyphenolic flavonoid compounds can block the initiation or reverse the promotion stage of multistep carcinogenesis. Quercetin is one of the most abundant flavonoids found in fruits and vegetables and has been shown to have multiple properties capable of reducing cell growth in cancer cells. Acute myeloid leukemia (AML) and myelodysplastic syndromes (MDS) therapy remains a challenge for hematologists worldwide, and the outcomes for patients with both disorders continue to be poor. This scenario indicates the increasing demand for innovative drugs and rational combinative therapies. Herein, we discuss the multitarget effects of the flavonoid quercetin, a naturally occurring flavonol, on AML and MDS.

## 1. Introduction

**Figure 1:**
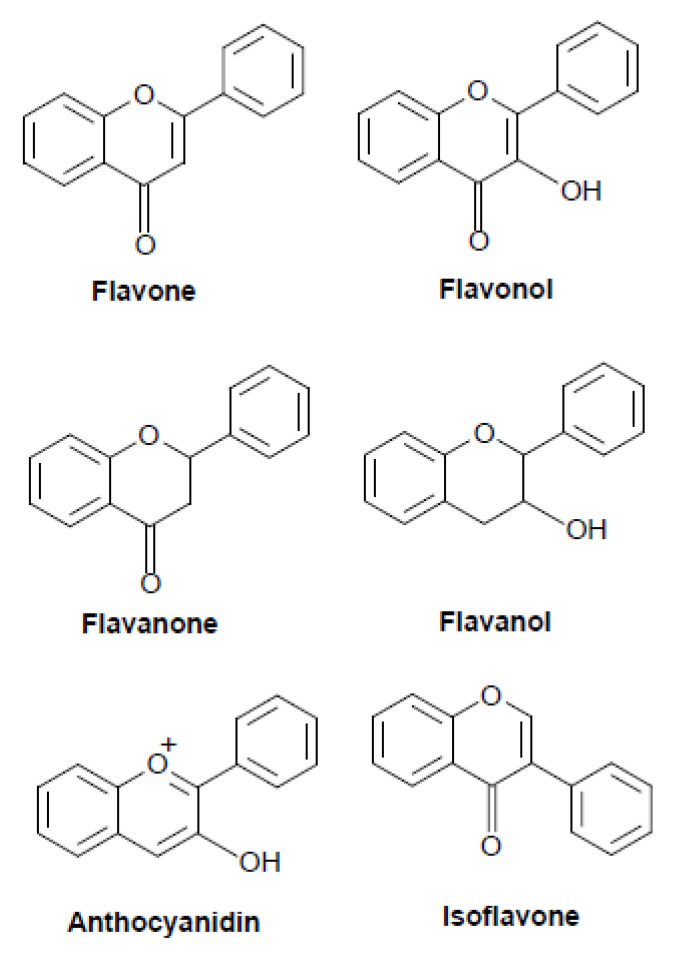
Structure of the major classes of flavonoids.

Flavonoids are ubiquitous groups of polyphenolic compounds present in natural products and plants such as fruits, vegetables, grains, bark, roots, stems, flowers, tea, and wine [1]. These natural substances have been shown to present promising chemopreventive and chemotherapeutic properties with multiple target interactions and multiple pathway regulation against various human cancers. Polyphenolic flavonoid compounds can block the initiation or reverse the promotion stage of multistep carcinogenesis [2]. Flavonoids are usually classified into six main subgroups: flavone (baicalein, apigenin), flavonol (fisetin, quercetin), flavanone (eriodyctiol, hesperidin), flavanol (catechin, epicatechin), anthocyanidin (cyaniding, apigeninidin), and isoflavone (daidzein, prunetin) (Figure 1).

**Figure 2:**
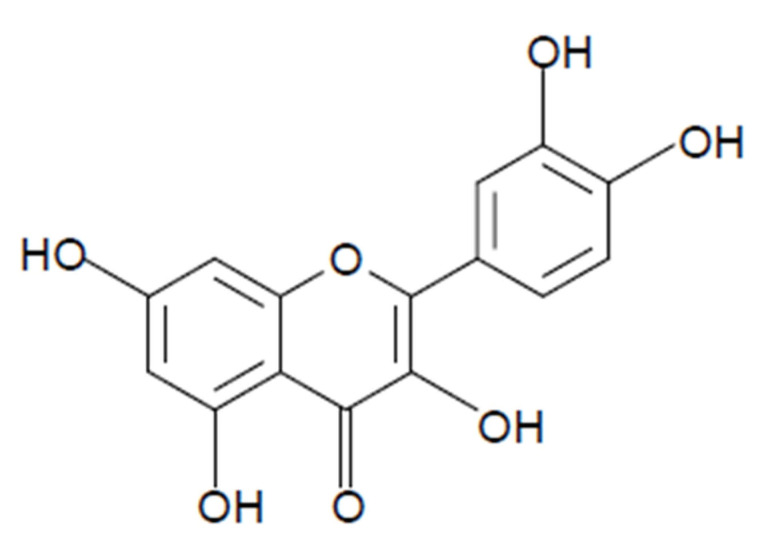
Structure of quercetin. Quercetin contains the basic structure of flavonoids: diphenylpropane (C6–C3–C6) with hydroxyl groups attached to the rings. The official nomenclature according to IUPAC is 2-(3,4-dihydroxyphenyl)-3,5,7-trihydroxy-4H-chromen-4-one. Other names include 5,7,3′,4′-flavon-3-ol, sophoretin, meletin, quercetine, xanthaurine, quercetol, quercitin, quertine, and flavin meletin. Chemical formula: C_15_H_10_O_7_.

Quercetin (3,3′,4′,5,7-pentahidroxiflavonol) is one of the most abundant flavonoids (Figure 2) belonging to the flavonol subgroup, found in fruits, vegetables, tea, and red wine [3,4]. Quercetin is also found in medicinal plants including *Allium cepa* (Liliaceae), *Allium fistulosum* (Amaryllidaceae), *Apium graveolens* (Apiaceae), *Asopargus officinalis* (Aspargaceae), *Camellia sinensis* (Theaceae), *Capparis spinosa* (Capparaceae), *Calamus scipionum* (Calamoidaceae), *Centella asiatica* (Apiaceae), *Coriandrum sativum* (Apiaceae), *Ginkgo biloba* (Ginkgoaceae), *Hypericum perforatum* (Hyperiaceae), *Hypericum hiricinum* (Clusiaceae), *Lactuca sativa* (Asteraceae), *Moringa oleifera* (Moringa), *Morus alba* (Moraceae), *Nasturtium officinale* (Brassicaceae), *Solanum lycopersicum* (Solanaceae), *Vitis vinifera* (Vitaceae), and *Sambucus canadensis* (Adoxaceae) [5,6,7]. Quercetin has anti-inflammatory, anticancer, cardiovascular protection, antiviral, antidiabetic, immunomodulatory, antiallergy, and antihypertensive therapeutic properties, in addition to gastroprotective effects. Quercetin is a yellow-colored crystalline insoluble solid substance and has a bitter taste. Quercetin is slightly soluble in alcohol, aqueous alkaline solutions, and glacial acetic acid [5].

**Figure 3:**
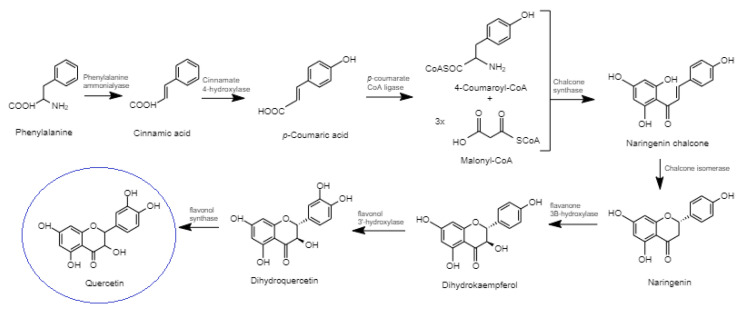
Quercetin biosynthesis.

Quercetin biosynthesis occurs though the phenylpropanoid metabolic pathway. Firstly, phenylalanine is converted to cinnamic acid; this reaction is catalyzed by the enzyme phenylalanine ammonialyase (Figure 3). Cinnamic acid undergoes the action of the enzyme cinnamate 4-hydroxylase to produce *p*-coumaric acid. This carboxylic group of synthesized *p*-coumaric acid undergoes ligation with CoA and produces 4-coumaroyl-CoA; this reaction is catalyzed by the enzyme p-coumarate:CoA ligase. Naringenin chalcone is produced by the enzyme chalcone synthase from one molecule of 4-coumaroyl-CoA and three molecules of malonyl-CoA to form the essential A and B rings of the flavonoid skeleton (i.e., C6–C3–C6). The chalcone isomerase synthesizes naringenin (a flavanone); this reaction enables the construction of the heterocyclic C ring. Flavanone 3β-hydroxylase undergoes hydroxylation of naringenin and produces dihydrokaempferol. Flavonol 3′-hydroxylase undergoes the hydroxylation reaction on dihydrokaempferol to produce dihydroquercetin. Finally, the activity of the enzyme flavonol synthase on dihydroquercetin catalyzes the biosynthesis of quercetin [8,9].

Quercetin has high bioavailability due to its aglycone structure, i.e., without a sugar group. The daily intake of quercetin in the diet is estimated to be 5–40 mg/day [2]; however, these values may be as high as 200–500 mg/day in individuals who consume large amounts of fruits and vegetables rich in quercetin (apples, onions, and tomatoes) [10]. In food, quercetin is found in a glycosylated pattern with bioavailability, which depends on the type of glycoside present in the molecule. After absorption, quercetin is metabolized in different organs, such as the intestine, colon, liver, and kidneys, where the molecule is conjugated to methyl, sulfate, and glucuronic acid [11]. The total amount of quercetin available in the plasma deriving from the diet is usually in the nanomolar concentration range (<100 nM); however, upon quercetin supplementation, this availability may increase to the high nanomolar or low micromolar range. For example, after 28 days of supplementation with 1 g/day of quercetin, concentrations of up to 1.5 μM have been observed [12,13].

In vivo studies reported that low doses of 50–500 mg/kg per day of quercetin do not cause relevant adverse effects or carcinogenic effects in animals [13]. In humans, a phase I clinical study recommended a dose of 1400 mg/m^2^, i.e., 2.5 g for subjects weighing 70 kg, administered via intravenous infusions for 3 weeks at weekly intervals. High doses exceeding 50 mg/kg (i.e., 3.5 g/70 kg) produced renal toxicity without signs of nephritis or obstructive uropathy [14]. Other studies found no adverse effects associated with oral administration of quercetin in a single dose of up to 4 g or after one month with 500 mg twice daily [15]. In 1999, the International Agency for Research on Cancer concluded that quercetin is not classified as carcinogenic to humans. In the United States and Europe, supplements of quercetin are commercially available, and the beneficial effects of quercetin supplements were reported from clinical trials [16].

Quercetin has been shown to have multiple properties capable of reducing cell growth in cancer cells due to a variety of biological activities including antioxidant, antiproliferative, apoptotic, and autophagic capacities.

Acute myeloid leukemia (AML) is an aggressive and malignant disorder of hematopoietic stem cells characterized by the clonal expansion of abnormally differentiated blasts of myeloid lineages, having unfavorable prognosis and short life expectancy [17]. Myelodysplastic syndromes (MDS) comprise a heterogeneous group of clonal hematopoietic neoplasms characterized by aberrant myeloid differentiation, ineffective hematopoiesis, refractory cytopenia, and increased rates of AML progression [18].

AML and MDS therapy remains a challenge for hematologists worldwide, and the outcomes of patients with both disorders remain poor. In MDS, the approval of azacitidine, decitabine, and lenalidomide over the last decade represented a major advance for this disease, despite the limited efficacy of these agents and most patients progressing within 2 years. Stem cell transplantation remains the only curative therapy; however, this option is associated with restricted efficacy and toxicity, and the lack or loss of response following standard therapies is related to dismal outcomes [18]. In AML, despite approximately 50% of patients successfully recovering from chemotherapy and bone marrow transplantation, elderly people are not capable of undergoing aggressive therapies, which demonstrates the urgent need for innovative drugs and rational combination therapies [19].

Herein, we discuss the multitarget effects of the flavonoid quercetin, a naturally occurring flavonol, on acute myeloid leukemia and myelodysplastic syndrome (Table 1).

## 2. Multitarget Effects of the Flavonoid Quercetin on Acute Myeloid Leukemia and Myelodysplastic Syndrome

### 2.1. Antiproliferative Activity

Quercetin inhibits the growth and proliferation of cell lines from different types of cancers (prostate, lung, breast, colon, and neoplasms) [20,37,38,39,40], and several mechanisms have been proposed to explain these effects. These studies have identified the ability of this compound to interact with specific regulatory proteins such as cyclins A, B, D and E; cyclin-dependent kinases (CDKs); and CDKs inhibitors (p21 and p27). Depending on the cell type, quercetin is able to block the G2/M or G1/S transition of the cell cycle.

The CDK activity regulator p21 is an important cell growth arrest mediator that inhibits cell entry into the G1 phase in response to DNA damage and hampers the re-entry of G2 cells into the S phase by blocking cyclin E-CDK2 mediated retinoblastoma (Rb) phosphorylation [41]. Rb is a direct substrate of CDKs and multiple mechanisms are known to potentially inhibit Rb phosphorylation, including proteasomal degradation [42] and inhibition of upstream Ras/RAF/MAPK or PI3K/Akt pathways [43,44].

Quercetin in vitro blocks cell-cycle progression at the G1 phase in P39 cells, a myeloid cell line derived from MDS-chronic myelomonocytic leukemia. Quercetin decreased the levels of CDK2, CDK6, cyclin D, cyclin E, cyclin A, and the phosphorylation of Rb accompanied by induction of p21 and p27 in these cells. In accordance, quercetin induced arrest in the G1 phase of the cell cycle in immunodeficient mice xenografted with P39 cells. The treatment of these mice with quercetin once every four days by intraperitoneal injection at 120 mg/kg body weight decreased the expression of phosphorylated Rb and the cyclin D and cyclin E proteins. Quercetin treatment also resulted in pronounced induction of p21 in xenografts [21]. In addition, pronounced ERK 1/2 and JNK phosphorylation in P39 cells and in P39 xenografts was induced. P39 cells treated with a combination of quercetin and selective inhibitors of ERK 1/2 and/or JNK (PD184352 or SP600125, respectively) decreased cells in the G1 phase, supporting the idea that ERK and JNK activation plays an important role in quercetin-induced G1 phase arrest in P39 cells [21].

Further studies have demonstrated that quercetin suppressed cell proliferation in the HL-60 cell line, derived from a 36-year-old woman with acute promyelocytic leukemia. Quercetin-induced G0/G1-phase arrest occurred [22,23] when expressions of cyclin-dependent kinases CDK2 and CDK4 were inhibited, and the CDK inhibitors, p16 and p21, were induced [23]. Quercetin also displayed antiproliferative activity against the HL-60 xenografts. After 3 weeks of quercetin treatment, a pronounced decrease in the protein levels of CDK2, CDK6, cyclin D, cyclin E and cyclin A were found. Quercetin treatment also resulted in Rb-phosphorylation loss [24].

The antiproliferative effect of quercetin was also demonstrated in the human promonocytic U937 cell line [20]. Quercetin induced antiproliferation and arrest of G2/M phase in these cells. An increase in the level of cyclin B in contrast to decreased levels of cyclin D, cyclin E, E2F1, and E2F2 was found in quercetin-treated U937 cells. Removal of quercetin from the cultures stimulated U937 cells to synchronously re-enter the cell cycle decreased the expression level of cyclin B, and increased the expression level of cyclin D and cyclin E, demonstrating that quercetin promotes reversible G2/M phase arrest. Quercetin further caused DNA fragmentation and increased the sub-G1 population in U937 cells [20].

### 2.2. Apoptotic Activity

The apoptotic effect of quercetin may result in the activation of multiple pathways in different types of cancer cell lines [25]. In vitro, quercetin led to pronounced apoptosis in the MDS-chronic myelomonocytic leukemia P39 cell line by modulating apoptotic cell death and stimulating the intrinsic and extrinsic apoptosis pathway [21]. Quercetin induced upregulation of the pro-apoptotic protein Bax and suppression of antiapoptotic proteins BcL-2, BcL-xL, and McL-1 in P39 cells. Furthermore, a decline in Rho123 fluorescence intensity and a release of cytochrome c from the mitochondria to the cytoplasm suggested that intracellular events related to P39 cell death caused by quercetin treatment are related to mitochondrial dysfunction and changes in the mitochondrial membrane potential (Δψm). In addition, significant enhancements in cleaved caspase-9, caspase-8, and caspase-3 were observed after quercetin treatment in P39 cells. Improvement in active caspase-8 and increased expression of FasL indicate that the extrinsic or death receptor pathway of apoptosis is activated by quercetin in P39 cells. The results obtained in vivo confirm these findings, since quercetin treatment resulted in the reduction in P39 cell viability and decreased tumor volume of P39 subcutaneously xenografted in immunodeficient mice. Quercetin caused apoptosis activation and decreased the expression of BcL2, BcL-xL, and McL-1 followed by increased Bax expression in P39 xenografts, as well as enhanced cleaved caspase-3 in P39 xenografts [21].

The apoptotic effect of quercetin was also studied in NOD/SCID mice xenografted with HL-60 AML cells. Quercetin reduced the expression of antiapoptotic proteins BcL-2, BcL-xL, and McL-1, but increased the expression of the proapoptotic protein BAX in xenografts. Induction of caspase-3 activation was further observed after quercetin treatment [24]. Yuan et al. [22] showed that quercetin caused apoptosis in HL-60 cells by decreasing the protein expression of PI3K and Bax, inhibiting Akt phosphorylation, decreasing the levels of BcL-2, and increasing the activation of caspase-2 and caspase-3 [22]. Quercetin induced apoptosis in HL-60 AML cells by increasing the activation of caspase-9, caspase-3, and cleaved poly ADP-ribose polymerase (PARP). Cleaved PARP protein levels were also increased in tumors obtained from HL-60 xenografts [23]. Niu et al. [26] demonstrated that quercetin induced apoptosis in HL-60 cells in a caspase-3-dependent manner by regulating the expression of downstream apoptotic proteins Bcl-2 and Bax [26]. Quercetin further potentiated the apoptosis induced by the glycolytic inhibitor 2-deoxy-d-glucose (2-DG) in HL-60 cells. Cotreatment with quercetin and 2-DG elicited the opening of mitochondria pore transition, which preceded the triggering of apoptosis [27]. Quercetin induced the activation of ERK, and the inhibition of ERK by an ERK inhibitor abolished the quercetin-induced cell apoptosis in HL-60 cells, as confirmed by the activation of caspase-8, caspase-9, caspase-3, PARP cleavage, and mitochondrial membrane depolarization in these AML cells. In vivo experiments corroborated these findings by demonstrating that quercetin reduced tumor growth through activating the ERK pathway and the subsequent cell apoptosis in HL-60 xenografts [28].

The ability of quercetin to induce apoptosis via the mitochondrial pathway was also demonstrated in human promonocytic U937 cells: loss of mitochondrial membrane potential prompted the release of cytochrome c in the cytoplasm with the activation of caspase-3 [20]. Cheng et al. [29] reported that the administration of quercetin led to pronounced apoptosis in the human leukemia cell line U937 by Mcl-1 downregulation, Bax conformational change, and mitochondrial translocation that triggered cytochrome c release. To prove quercetin’s effects, knockdown of Bax by siRNA was found to reverse quercetin-induced apoptosis and abolish the apoptosis and activation of caspase. Knockdown of Mcl-1 by siRNA improved Bax activation and translocation, and cell death induced by quercetin. Furthermore, in vivo administration of quercetin mitigated tumor growth and increased TUNEL-positive apoptotic cells in tumor sections of U937 xenografts. Quercetin also increased the expression of the pro-apoptotic protein Bax and inhibited the antiapoptotic McL-1 protein in xenografts [29]. Quercetin in combination with shHSP27 synergistically induced apoptosis by decreasing the Bcl2-to-Bax ratio in U937 cells [30]. The apoptosis induced in U937 cells occurred by downregulation of Mcl-1 acting directly or indirectly on mRNA stability and protein degradation [31]. Quercetin was also reported to cause the induction of PARP cleavage in the three AML cell lines: THP-1, MV4-11, and U937 [28]. Quercetin induced cell death by downregulating VEGRF2 and PI3K/Akt signaling; these pathways were related to quercetin’s action on mitochondria and BCL2 proteins [36]. Increased VEGRF2 is related to chemotherapy resistance in human leukemia cells, and VEGRF2 reduction sensitizes AML cells to chemotherapy treatment, leading to an increase in mitochondrial mass and oxidative stress [45]. These findings reveal quercetin as a promising compound targeting VEGRF2. These data suggest that the ability of quercetin to induce apoptosis (both intrinsically and extrinsically) into cancer cells establishes quercetin, without a doubt, as a promising molecule in the field of oncology.

### 2.3. Autophagic Activity

Quercetin demonstrated autophagic activity on both acute myeloid leukemia and myelodysplastic syndrome cell lines. Quercetin was found to induce autophagy and cell death [46] and, apparently, autophagy activation is an attempt to rescue cells from induced apoptosis [47]. This flavonol may induce sirtuins [48], reactive oxygen species (ROS) production [49], and JNK 1/2; Mek/ERK [50], all of which are capable of influencing the regulation of the autophagic process. Another target is represented by PI3K pathway proteins, which may positively or negatively affect autophagic regulation. In addition, quercetin assists in the accumulation of hypoxia-induced factor (HIF)-1 alpha, which represses mTOR signaling and induces the expression of BNIP3/BNIP3 ligand, supporting the rupture of the Beclin-1/BcL-2 (BcL-xL) complex for activation of Atg7 and Atg12-Atg5 and the cleavage of LC3 [47]. Conversely, quercetin may further inhibit autophagic flow. This result was obtained by the inhibition of PI3K class III and the NF-κB pathway (which may inhibit or induce autophagy depending on the context) [51]. Another interesting property of quercetin is that, when in combination with other substances, it may potentiate autophagic and apoptotic effects [52].

Quercetin triggered autophagy both in vitro and in vivo in P39 cells in the MDS-derived leukemia cell line [21]. High acidic vesicular organelle formation was detected in quercetin-treated P39 cells. Quercetin increased the expressions of light chain 3 (LC3)-II, PI3K, Beclin-1, Atg5-Atg12, and Atg7 in P39 cells. The inhibition of autophagy in P39 cells using chloroquine (endossomal acidification inhibitor) triggered apoptosis but did not alter the modulation in the G1 phase, suggesting that quercetin-induced autophagy plays a protective role against apoptotic cell death in P39 cells but is not associated with the cell cycle [21]. In accordance with the in vitro results, quercetin increased the expressions of PI3K, Beclin-1, Atg5- Atg-12, and Atg7 in P39 xenografts. Increased cleaved LC3 was further detected in the tumor sections of xenografts by immunohistochemistry. Quercetin additionally dephosphorylated Akt and mTOR in P39 cells and P39 xenografts, underlining the functional importance of Akt–mTOR signaling in quercetin-mediated protective autophagy in P39 cells [21]. Quercetin was described to affect both mTOR activity and activation of PI3K/Akt signaling pathway, giving quercetin the advantage of functioning as a dual-specific mTOR/PI3K inhibitor. Akt-mTOR signaling is a frequently hyperactivated pathway in cancers and, as a key regulator of homeostasis, controls the essential pathways leading to cell growth, protein synthesis, and the autophagic process [53].

The induction of autophagy by quercetin on HL-60 AML xenografts was additionally described [24]. Quercetin increased the expressions of PI3K, Beclin-1, ATG7, and ATG12-ATG5 in HL-60 xenografts, and caused the pronounced induction of the LC3-I to LC3-II switch [24]. Chang et al. [23] reported the effect of quercetin on autophagy both in vitro and in vivo in the HL-60 AML cell line. Increased expression of LC3-II, decreased expression of p62, and formation of acidic vesicular organelles were found after quercetin treatment [23]. Decreased protein expression of PI3K and phosphorylated Akt were reported in the HL-60 cell line after quercetin treatment [22].

### 2.4. Antioxidant Activity

Quercetin is considered one of the most prominent dietary antioxidants [11]. The antioxidant effect of quercetin has been extensively described [54]. Quercetin is a potent scavenger of ROS, including superoxide (O^2−^), and reactive nitrogen species (RNS) such as nitric oxide (NO) and peroxynitrite (ONOO–). These antioxidative capacities of quercetin are attributed to the presence of two pharmacophores within the molecule that have the optimal configuration for free radical scavenging: the catechol group in the B ring and the OH group at position 3 of the AC ring [11].

**Figure 4:**
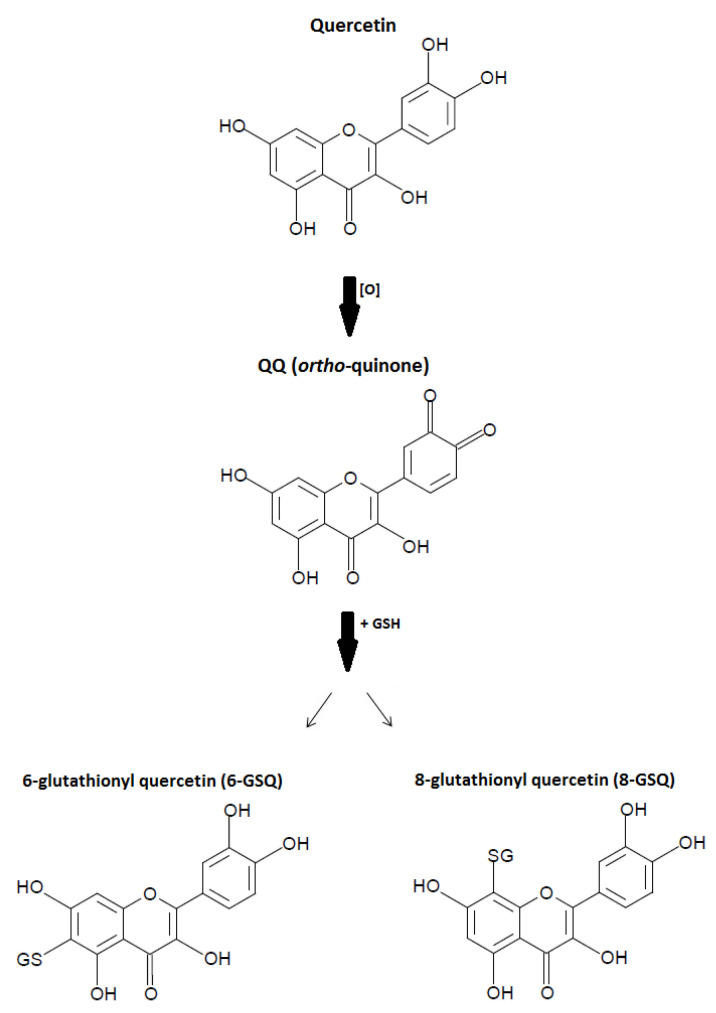
Antioxidative mechanism of quercetin. Quercetin is oxidized into oxidation products (QQ: ortho-quinone) and reacts with glutathione (GSH) to form 6-glutathionyl quercetin (6-GSQ) and 8-GSQ.

During antioxidative activities, quercetin becomes oxidized into oxidation products (QQ: ortho-quinone) and reacts with glutathione (GSH) to form 6-glutathionyl quercetin (6-GSQ) and 8-GSQ (Figure 4). Oxidation products such as semiquinone radicals and quinones have been well-described to display various toxic effects in vitro only due to their ability to arylate protein thiols [11]. The antioxidative mechanism of quercetin occurs primarily with the O_2_ bond yielding the ortho-quinone and H_2_O_2_ radicals. Quercetin further reacts with H_2_O_2_ in the presence of peroxidase, decreasing H_2_O_2_ levels. In addition, the antioxidant capacity of quercetin depends on the availability of intracellular glutathione (GSH).

The antioxidative aspect of quercetin was associated with the amelioration of adverse side effects derived from cancer therapy, which is considered a suitable strategy for the prevention and treatment of cancer [55]. This property of quercetin was demonstrated in both acute myeloid leukemia and myelodysplastic syndrome.

Quercetin decreased ROS levels in P39 MDS-derived leukemia cells induced by ter-butylhydroperoxide [21]. The antioxidative activity of quercetin in P39 cells could be explained by its capacity to interact with important antioxidant cellular defense systems, such as the Nrf2/Keap complex. This complex is connected with apoptotic pathways through the regulation of proteins from the Bcl-2 family. Keap1 appears to restrain and destabilize Bcl-2 and decrease Bcl-2:Bax heterodimers, facilitating cancer cells apoptosis [56].

Quercetin further reduced ROS and NO in LPS-stimulated human THP-1 acute monocytic leukemia cells [32]. In contrast, quercetin was shown to induce the cell death of HL-60 AML cells in vitro and in vivo through the induction of intracellular oxidative stress following activation of ERK. The authors demonstrated that mitochondrial superoxide and intracellular peroxide levels in HL-60 AML cells and HL-60 xenografts were higher after quercetin treatment [28]. In another study, quercetin caused disparate effects on ROS generation in HL60 cells, and did not affect GSH levels [27]. In a human acute promyelocytic leukemia NB4 cell line, quercetin increased the levels of Nrf2 in the cytosol, reducing them in the nucleus [33].

### 2.5. Epigenetic Modulation

An important feature of quercetin was recently reported: the ability to affect epigenetic regulators. Studies evaluating the epigenetic effects of quercetin in MDS are not available. In AML, quercetin induced cell death in HL-60 and U937 leukemia cell lines by targeting the epigenetic regulators of pro-apoptotic genes [34]. The results showed that quercetin treatment abolishes DNA methyltransferase (DNMT) 1 and DNMT3a expressions partly due to STAT-3 regulation. Downregulation of class I histone deacetylases (HDACs) by quercetin was also detected. Treatment of cells with MG132, a proteasome inhibitor, in combination with quercetin prevented the degradation of class I HDACs compared to cells treated with quercetin alone, indicating increased proteasome degradation of class I HDACs by quercetin. Demethylation of the pro-apoptotic BCL2L11 and DAPK1 genes in a dose- and time-dependent manner was observed after quercetin treatment. Moreover, quercetin caused a global increase in acetylated histone 3 and histone 4, resulting in three- to ten-fold increases in the promoter regions of DAPK1, BCL2L11, BAX, APAF1, BNIP3, and BNIP3L. The increase in the mRNA levels of all these genes induced by quercetin corroborates these findings [34]. Briefly, the induction of apoptosis by quercetin appears to be related to the influence of this flavonoid on DNA demethylation activity, HDAC inhibition, and the enrichment in H3ac and H4ac in the promoter regions of genes involved in the apoptosis pathway, leading to their transcription activation.

Induction of FasL-related apoptosis by quercetin was described by transactivation through activation of c-jun/AP-1 and promotion of histone H3 acetylation in human HL-60 leukemia cells [35].

## 3. Conclusions

**Figure 5:**
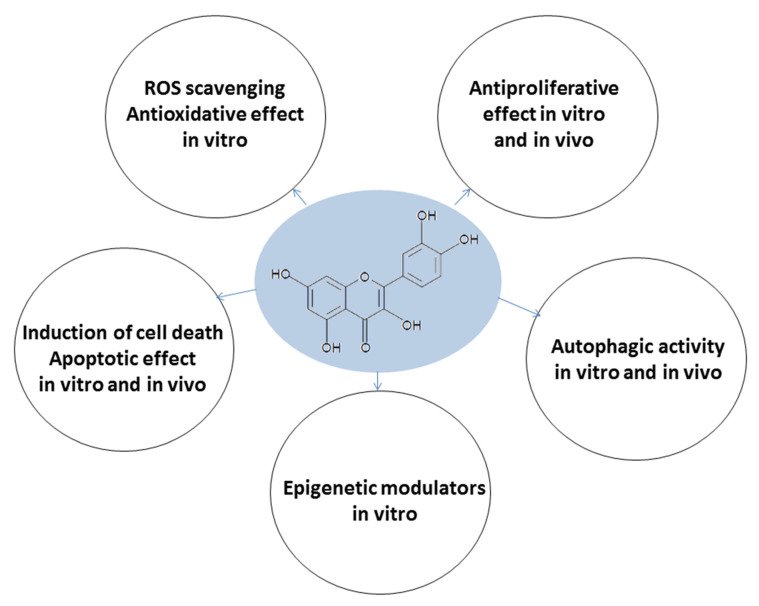
Multitarget effects of quercetin on acute myeloid leukemia and myelodysplastic syndrome.

The natural polyphenolic flavonoid compound quercetin has emerged as a molecule possessing multiple properties (Figure 5). Quercetin has been proven to be an excellent antioxidant that also possesses antiproliferative, apoptotic, and autophagy capacities in AML in vitro and in vivo, in addition to exerting effects on epigenetic modulation. The effects on MDS are poorly understood as few studies have been performed. Clinical studies evaluating quercetin’s effects on AML and MDS patients are not available. However, we conclude that quercetin has the potential to induce cell death in human AML and MDS-derived leukemia cell lines, both in vitro and in vivo, and that this capacity is related to a mechanism involving multitarget cooperation. The ability of this flavonoid to interfere with different mechanisms involved in cancer development indicates quercetin as an alternative for acute myeloid leukemia and myelodysplastic syndrome.

## Figures and Tables

**Table 1:** Summary of activities for quercetin in AML and SMD.

Activity	Cancer Cell Type	Notes	Reference
Antiproliferative, apoptotic	U937	In vitro	[20]
Antiproliferative, apoptotic, autophagic, antioxidant	P39	In vitro, xenograft	[21]
Antiproliferative, apoptotic, autophagic, antioxidant	HL-60	In vitro	[22]
Antiproliferative, apoptotic, autophagic, antioxidant	HL-60	In vitro, xenograft	[23]
Antiproliferative, apoptotic, autophagic, antioxidant	HL-60	xenograft	[24]
Apoptotic	U937	In vitro	[25]
Apoptotic	HL-60	In vitro	[26]
Apoptotic, antioxidant	HL-60, THP-1, NB4	In vitro	[27]
Apoptotic, antioxidant	HL-60, THP-1, MV4-11, U937	In vitro, xenograft	[28]
Apoptotic	U937	In vitro, xenograft	[29]
Apoptotic	U937	In vitro	[30]
Apoptotic	U937	In vitro	[31]
Antioxidant	THP-1	In vitro	[32]
Antioxidant	NB4	In vitro	[33]
Epigenetic modulation	HL-60, U937	In vitro, xenograft	[34]
Epigenetic modulation	HL-60	In vitro	[35]
Apoptotic	MV4-11, HL-60	In vitro	[36]

## Data Availability

Not applicable.
